# Spontaneous approaches of divers by free-ranging orcas (*Orcinus orca*): age- and sex-differences in exploratory behaviours and visual laterality

**DOI:** 10.1038/s41598-017-11488-3

**Published:** 2017-09-07

**Authors:** Stéphanie Chanvallon, Catherine Blois-Heulin, Pierre Robert de Latour, Alban Lemasson

**Affiliations:** 1 Université de Rennes 1, Ethologie animale et humaine – EthoS, UMR 6552 – CNRS – Université Caen Normandie, Station Biologique, 35380 Paimpont, France; 2 USEA, Orques Sans Frontières, 40280 Bretagne-de-Marsan, France

## Abstract

Running comparative studies of laterality in mammals is a way to deepen our understanding of the evolution of the brain hemisphere functions. Studies on vision highlighted a possible task-sharing between hemispheres depending on the characteristics of the observers, the nature of the observed stimulus and the context of the observation, a phenomenon that could go beyond the monitoring of conspecifics. Cetaceans are predators that adapted to an aquatic habitat and display a clear crossing of fibers to the side of the brain opposite the eye of origin. Here, we analysed the interactions between humans and cetaceans when free-ranging orcas approach divers. Our study concentrated on the spontaneous exploratory behaviours of divers by orcas depending on their age and sex, and on the possible expression of a visual laterality. The results showed a significant preference for the use of the left eye but exclusively in adult females. Adult males had a more sustained attention than adult females, marked by a higher spatial proximity to divers, slower approaches and longer look durations. Adult females, probably more cautious, explored from the distance and more furtively. Our findings support a possible link between attentional/motivational states and visual laterality in mammals.

## Introduction

Recent studies suggest that brain laterality is much older than we thought until now and certainly not unique to humans^1, 2^. We actually find behavioural descriptions of motor and perceptual laterality in nearly all the groups of vertebrates as well as in some invertebrates. Visual laterality has notably been documented in a large number of species (i.e. Fish:^3, 4^; Amphibians:^5, 6^; Reptiles:^7, 8^; Birds:^9, 10^; Mammals:^11–15^; Invertebrates:^16, 17^), but rarely in large marine mammals because of the difficulty of observing them in the field and their relative rarity in captivity. According to Karenina *et al*.^18^, when individuals need to coordinate their behaviour with others, the uniformity of preferential sides inside a population is advantageous. For many animal species, though a consensus appears around the dominant use of one eye over the other one (and therefore the dominant processing by a given hemisphere), the variations regarding which eye (i.e. left or right) is used preferentially are important and seem to depend on the nature of the observed stimulus (e.g. novelty, valence and complexity^4, 19–26^, as well as on the individual characteristics of the observer (e.g. vigilance state and level of stress:^27^; age:^28^) and on the social context of the interaction^29–32^. Laterality at population or group levels is pointed out in different contexts such as foraging for food^23, 24^, antipredator^33^ or social vigilance^29, 34, 35^, as well as environmental exploration^13, 22^.

Cetaceans, contrasting to what was once believed, because of their often dark natural habitat (i.e. deep water) and developed echolocation^36^, have good vision in the air as underwater^37^. Furthermore, cetaceans have eyes in a semi lateral position with a near total crossing of optical nerves (left eye - right hemisphere)^38^. A few studies actually underline the existence of a possible visual laterality in cetaceans, even though the factors determining the expression of this laterality are still not very well known. For example, in free-ranging belugas (*Delphinapterus leucas*) and orcas (*Orcinus orca*), the juvenile is often positioned on the right side when it swims near its mother, maintaining a visual contact with its left eye^18, 39^. This preference for a lateralized swim position was recently confirmed with a beluga population in managed care^40^. Nevertheless, differences are found between social and non-social monitoring. When hunting, orcas attack their prey by placing them in their right visual field, whereas no lateral bias is found when they jump out of water and fall back on their side^41^. The right hemisphere of cetaceans would thus play a predominant role for social interactions^18, 39, 42^, whereas in response to non-social or non-familiar stimuli, a predominance of the left hemisphere can be found^26^.

In captivity, the left eye was preferentially used by bottlenose dolphins (*Tursiops truncatus*) to observe a human being, familiar or not, approaching the pool^17^. The individual left eye lateralized preferences were later confirmed by studies with captive belugas and pacific white-sided dolphins (*Lagenorhynchus obliquidens*)^43^. In the same experimental conditions, when the human being was replaced by an object, the degree of familiarity with the observed item was important. Indeed, Blois-Heulin *et al*.^22^ found that, while non-familiar objects are observed preferentially with the right eye, the left eye is used to look at very familiar (previously manipulated) objects. Both eyes are used indiscriminately for intermediary items, i.e. non-familiar objects which have become to some extent familiar from previous observations but never manipulated. All in all, the mother, the human (assimilated to the one that is providing care and food) and the toys in captivity are preferentially processed by the right hemisphere, whereas prey and new objects are processed by the left hemisphere. Interestingly, the degree of familiarity with the human or object observed also influenced gaze duration in several cetacean species^21, 43–45^. The question thus remains open as to whether or not the observer’s attention and motivational state may be a determining factor. More studies are now needed, notably with free-ranging cetaceans, and the cases of human – orca spontaneous encounters is particularly interesting because the former is a potential prey for these animals.

Orcas show numerous and complex capacities of social coordination, which involve both communication and cognition. They indeed show a great diversity of innovated and learned foraging techniques and feeding cooperation strategies^46–48^, individual and matrilineal acoustic signatures with dialectal cultural variations^47–49^ and a long period of maternal dependence for the single offspring^50^. Orcas are also curious animals characterized by a highly developed neophilia in captivity^51^ and frequent manipulation of various objects in the wild^52^. Recent studies have described cases of play behaviour innovations in resident orcas living in the U.S. west coast^46, 53^.

In several mammal species, innovations, risk-taking and exploratory behaviours vary with the age and sex of the individual (e.g. rats and primates:^54–57^). In captive dolphins, youngsters are generally more curious than adults^58^, and 80% of the new behaviours observed in plays come from juveniles^59^. However, Baird *et al*.^60^ found no influence of age and sex in diving rates in orcas, but male adults would dive more often during the day than female adults. The question of exploratory behaviour regarding age, sex, as far as we know, has never been studied in free-ranging orcas. Previous studies in cetaceans (e.g. dolphins and belugas) have shown that gaze duration (and possibly swimming speed and distance of approach) plays a key role during exploration of familiar and unfamiliar humans and objects^21, 43–45^. Although the exploratory behaviours of cetaceans have still been poorly described in the literature, particular behaviours like head and body movements, some being associated with vocalisations (notably echolocation), have also been previously described in this context^61, 62^.

How humans are perceived by free-ranging orcas is a current hot topic, notably because whale-watching and swimming-with-whales ecotouristic sites are multiplying. To this day, little research has been conducted scientifically about the nature of human - orca spontaneous encounters in the wild and in the diversity of associated behaviours. Orcas’ interest is expressed by behaviours like following boats and many spontaneous interactions with humans (cetologists, fishermen, tourists), including play and even actions of supposed cooperation with humans in their fishing activities^63^. In the wild, orcas are totally free of their movements and voluntarily approach divers. By using underwater videos, made in Norway, we therefore concentrated on gazes and other behaviours in the context of spontaneous approaches of divers by orcas. We studied an area where diving is organised every year. We have analysed the influence of individual’s age and sex on the behaviours displayed during these approaches and tested a possible eye-preference during visual monitoring.

## Results

**Figure 1:**
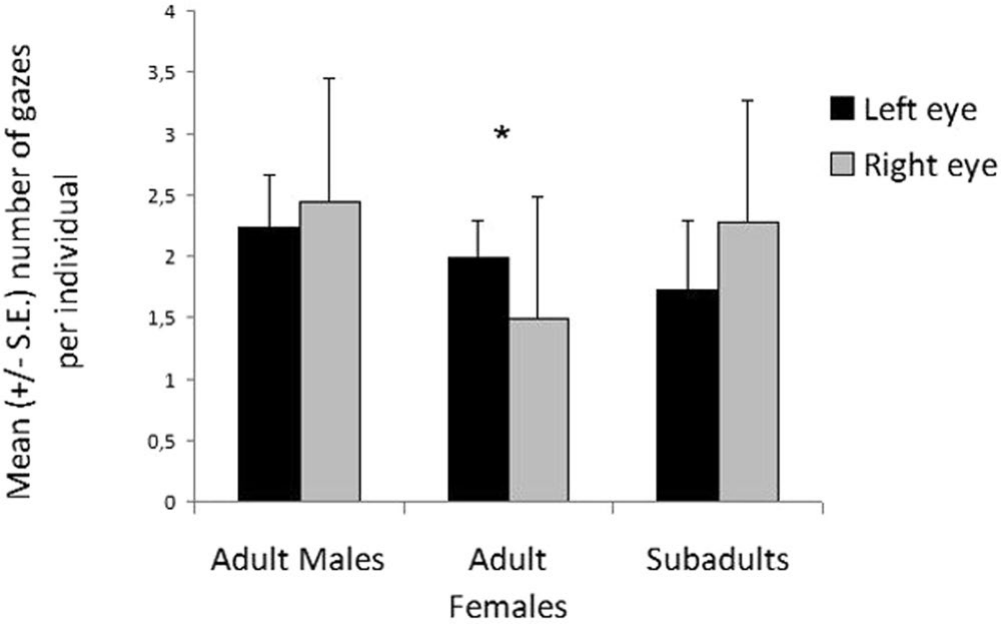
Preferential use of the right (grey bars) or left (black bars) eye by orcas according to their sex and age. Wilcoxon test: *p < 0.05.

Among all approaches sampled, 24%, 61.3% and 14.7% consisted of adult males, adult females and subadults respectively. Monocular gazes were clearly the majority (82.5%) of all gazes sampled. Nevertheless, the eye preferentially chosen by the different individuals varied with their age and sex. Thus, while adult males (Wilcoxon matched-paired test, *N* = 18, *Qobs* = 40, *p* = 0.97) and subadults (*N* = 11, *Qobs* = 7.5, *p* = 1) did not preferentially use one eye over the other, adult females used their left eye more frequently (N = 60, *Qobs* = 191, *p* = 0.04) (Fig. 1).

In most cases (86%), the “approach consisted in a progressive movement towards the divers and more rarely (14%) in a “passing by” movement (i.e. quasi-linear trajectory, constant distance with the divers). Moreover, orcas approached preferentially from the front or below (98%) and in very rare cases from the back (2%). After having excluded the rare approaches from behind, we found that all units were relatively frequent (i.e. from the right side 39%, from the left side 29%, facing 14%, just under 10%, from deep water 8%).

However, certain types of approach were more frequently observed than others in the three age-sex classes of individuals (see all statistical intra-class comparisons in Table 1). First, concerning the social context and the minimum distance of approach, we found that all individuals approached significantly more often alone than accompanied, and more often close than far. However, while adult females approached more often close than very close, no difference was found for adult males and subadults. Second, concerning visual monitoring and swimming speed, while adult females and subadults displayed more short than long gazes, no difference was found for adult males. This has to be related to some extent to the swimming speed that was more frequently slow than fast in adult males but equally slow and fast in both adult females and subadults. Third, concerning head movements, while adult males displayed more horizontal than vertical movements, both types were equally observed in adult females and subadults. Fourth, all individuals approached significantly more often silently than accompanied by whistle calls. Finally, two behaviours were found only in adult females and subadults, and not in adult males: belly presentation (adult females 6.7%, subadults 18% of times), and body rotation (adult females 16.1%, subadults 16.4%).

**Table 1:** Approach characteristics of the different age-sex classes.

Comparisons	Adult females	Adult males	Subadults
Alone *versus* with another orca(s)	3.05 ± 0.06/1.16 ± 0.02	4.61 ± 0.65/0.88 ± 0.37	4.72 ± 2.04/0.72 ± 0.54
	N = 56 z = 3.32	N = 17 z = 5.51	N = 11 z = 2.93
	**p** = **0**.**0009**	**p** = **0**.**0007**	**p** = **0**.**003**
Close *versus* Far	3.86 ± 0.07/0.3 ± 0	5.11 ± 0.83/0.2 ± 0.1	5.45 ± 2.67/0.1 ± 0.09
	N = 56 z = 6.23	N = 18 z = 3.62	N = 11 z = 2.93
	**p** = **0**.**00001**	**p** = **0**.**0003**	**p** = **0**.**003**
Very close *versus* Close	0.96 ± 0.03/2.9 ± 0	3.11 ± 0.57/2.05 ± 0.59	2.91 ± 2.25/2.54 ± 0.55
	N = 54 z = 4.89	N = 15 z = 1.47	N = 10 z = 1.68
	**p** < **0**.**0001**	**p** = 0.14	p = 0.09
Short *versus* Long gaze	2.71 ± 0.04/1.46 ± 0.04	2.16 ± 0.61/3.33 ± 0.56	3.72 ± 1.84/1.63 ± 0.86
	N = 40 z = 3.91	N = 16 z = 1.75	N = 9 z = 2.07
	**p** = **0**.**00009**	p = 0.08	**p** = **0**.**04**
Slow *versus* Fast swimming	4.25 ± 0.07/1.87 ± 0.03	4.22 ± 0.61/1.11 ± 0.43	4.45 ± 1.34/4.49 ± 1.35
	N = 52 z = 1.48	N = 17 z = 3.40	N = 10 z = 0.96
	p = 0.14	**p** = **0**.**0006**	p = 0.33
Horizontal *versus* Vertical head movement	0.36 ± 0.01/0.25 ± 0.01	0.5 ± 0.18/0.22 ± 0.10	0.36 ± 0.36/0.45 ± 0.36
	N = 16 z = 0.98	N = 6 z = 2.20	N = 1
	p = 0.33	**p** = **0**.**03**	
Whistling *versus* Silence	1.05 ± 0.27/3.13 ± 0.50	1.27 ± 0.40/4.22 ± 0.65	1.83 ± 1.05/8.33 ± 4.41
	N = 55 z = 4.47	N = 16 z = 2	N = 11 z = **3**.**059**
	**p** = **0**.**00008**	**p** = **0**.**0006**	**p** = **0**.**003**

In each cell: First line – mean number of occurrences (+/− standard error) for the two compared behavioural units; Second and third lines – Number of individuals taken into account in the statistical analysis (equal scores excluded), z score and p value of the Wilcoxon test (significances in bold). NA: test not applicable.

**Figure 2:**
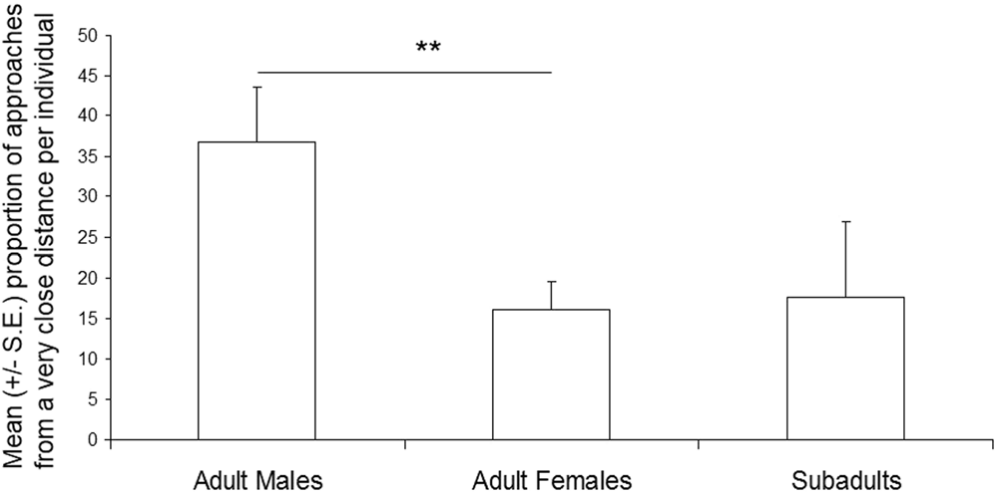
Variation of the individual percentage of approaches (mean +/− s.e.) from a very close distance with the age and sex of the orca. Mann-Whitney test: **p = 0.002.

**Figure 3:**
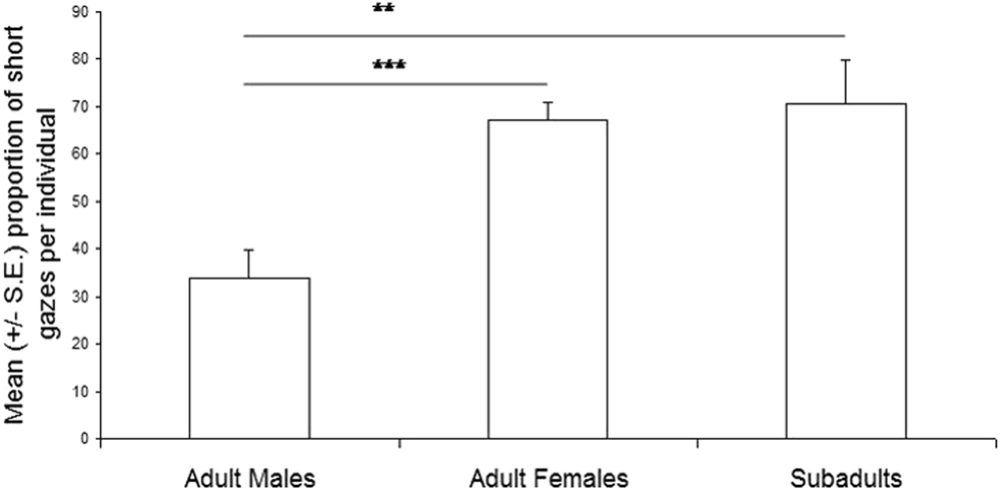
Variation of the individual percentage of short gazes (mean +/− s.e.) with the age and sex of the orca. Mann-Whitney test: ***p = 0.0001, **p = 0.004.

The above differences were confirmed for some of the measured variables when running comparisons between adult males, adult females and subadults (inter-classes comparisons). Indeed, adult males approached significantly the diver at a shorter distance than adult females (Mann-Whitney test, n1 = 18 n2 = 60, z = −3.11, *p* = 0.002), but only a trend was found with subadults (n1 = 18 n2 = 11, z = 1.77, *p* = 0.08) (Fig. 2). Also, adult females and subadults did not differ in the proportion of times they approached at a very close distance (z = 0.01, *p* = 0.99). Moreover, adult males displayed significantly fewer short gazes than adult females (z = 4.05, *p* = 0.0001) and than subadults (z = −2.89; *p* = 0.004), whereas adult males and subadults did not differ (z = −0.72; *p* = 0.47) (Fig. 3).

**Figure 4:**
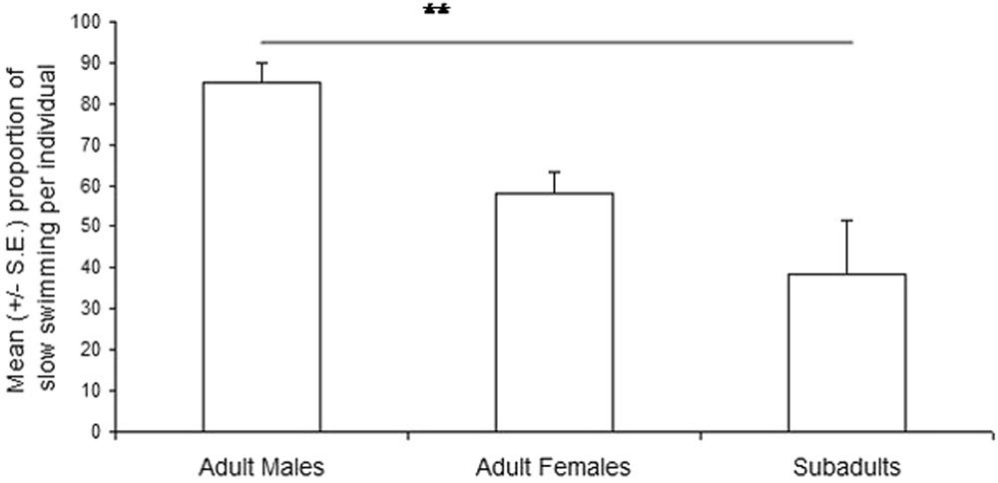
Variation of the individual percentage of slow swimming (mean +/− s.e.) with the age and sex of the orca. Mann-Whitney test: **p = 0.007.

The swimming speed was also significantly slower in adult males than in subadults (z = 2.29, *p* = 0.007), while adult females’ speed did not differ (when applying Bonferroni correction) with those of adult males (z = −2.001, *p* = 0.045) and subadults (z = 1.35, *p* = 0.18) (Fig. 4).

No influence of age and sex was found for the proportion of solitary approaches (Kruskall-Wallis test: H(2.89) = 2.69, p = 0.26; males 88.56 ± 4.10%, females 69.91 ± 5.26% and subadults 93.39 ± 3.57%), horizontal head movements (H(2.89) = 3.68 p = 0.16; males 34.72 ± 10.91%, females 20.97 ± 4.98% and subadults 4.55 ± 4.55%), whistling approaches (H(2.89) = 0.74, p = 0.69; males 34.72 ± 5.25%, females 22.51 ± 4.23% and subadults 12.14 ± 4.37%).

In order to better understand the context of whistling, we tested whether certain variables were more often associated than others. Indeed, we found that while there were as many approaches with whistles when the orca approached alone or not (Wilcoxon matched-paired test: *N* = 83, *Q* = 1806, *p* = 0.743), there were significantly more approaches with whistles when the swimming speed was slow than fast (*N* = 83, *Q* = 2478, *p* = 0.0001).

## Discussion

This first investigation of the nature of the exploratory behaviour of free-ranging orcas when approaching human divers shows two important findings. First, adult females prefer to visually monitor humans with their left eye than with their right eye, but no eye preference was found in adult males and subadults. Second, the approach behaviours differed significantly between different individuals; adult males demonstrated stronger attention, as well as potentially greater curiosity (shorter distance, slower swimming and longer gazes) than adult females and subadults, the latter being possibly more cautious.

The visual laterality in favour of the left eye is in accordance with the previous findings on social interactions in wild and captive cetaceans^18, 39^ and with captive dolphins when looking at humans^21^ and at familiar toys^22^. It therefore confirms the dominance of the right hemisphere in the visual exploration of some salient and familiar items by cetaceans (see also ref. 64) and allows us to support the hypothesis that the human diver is a source of interest for the free-ranging orcas habituated to their presence (for about 18 years). This hypothesis is also supported by the fact that no aggressiveness or sudden withdrawal has been noticed.

However, not all individuals preferred a left-side gazing in our study. Several non-mutually exclusive hypotheses may explain the individual variations observed. First, it is possible that laterality is more pronounced in older individuals than younger ones, due to their experiences with those unusual encounters, which would account for the absence of laterality in subadults. Indeed, the strength of laterality in animals is frequently related to the degree of familiarity with the observed item^22^. This is also supported by the fact that more adult females are in contact with divers than adult males in this area, and thus more experienced with human encounters. Over two consecutive years, the number of approaches by adult females was 4.5 times higher than the number of approaches by adult males. Second, it is possible that the emotional state of male and female adults differed in our study, as this is another factor of variation in humans and animals for both strength and direction of laterality^65– 67^. The fact that females generally keep a greater distance than males here and only observe humans briefly could be the sign of cautiousness in females that, while approaching, would remain more vigilant. Third, it is very likely that there is a link between the direction of the laterality and the degree of attention of the observer, as it was found earlier in birds^68^. Males, being more attentive, perhaps integrate more details, thus mobilizing their two hemispheres. The left hemisphere is known to play a key role for detailed visual exploration in various animal species^69–73^. Fourth, the hormonal difference between males and females may also be responsible for the sex-dependent laterality observed here. A study on testosterone-treated chicks found that an effect of testosterone was greater reliance on the frontal visual field^74^, which could be a possible explanation for the absence of lateral bias due to a stronger collaboration between the two hemispheres in male orcas. Fifth, differences in cognitive abilities between males and females could explain the sex-dependent laterality found in our study. In humans, females perform better than males in various memory tasks^75, 76^. This female superiority would be explained by a more effective use of the abilities of the right hemisphere for accessing memory of patterns made up of multiple items^2^.

Also in our study, adult males approached less often than adult females; this is probably due simply to their smaller number in the overall population. The fact that males approached divers at a closer distance and for longer exploration sequences is an important point of discussion. One abovementioned explanation is that males may be more curious. Unfortunately, to our knowledge, there is no such “personality type” study done with cetaceans that could confirm this hypothesis. However, sex-specific responses to human presence have been found earlier in cetaceans. Williams *et al*.^77^ studied the habituation to human activities by orcas in Johnson Detroits (British Columbia). They found that the animal’s reaction to boats changed in a sex-specific way with time. While females tended to accelerate their swimming speed and increase the angle of their diving routes, males kept their swimming speed constant with less predictable trajectory. This said, the fact that females approached less closely in our study does not mean that they did not “observe”; cetaceans may use echolocation to complete their visual exploration^36, 78^. It is possible that females prioritized long-distance echolocation over short-distance eye observation. In line with this, vertical head movements, typically associated with echolocation^61^, were particularly frequent in adult females. Scheer *et al*.^61^ also described this echolocation behaviour directed to humans at a 20-meter distance in free-ranging pilot whales (*Globicephala macrorhynchus*). As Blois-Heulin *et al*.^22^, we suppose that echolocation, associated to a binocular vision, completes the amount of information collected on objects, facilitating the construction of its representation.

The approaches described here are signs of a certain curiosity, and possibly attempts to initiate interactions with divers. The fact that the vast majority of the approaches come from the front clearly indicates that animals behave to remain visible. Scheer *et al*.^61^ with free-ranging pilot whales and Duroyon^62^ with captive bottlenose dolphins (*Tursiops truncatus*) have also described behaviours like belly presentation, body rotation, horizontal head movements that were clearly associated with non-agonistic encounters and interaction initiations. To our knowledge, the function of whistling in this context has never been studied, but it is intriguing to find more calls associated with closer and longer approaches. This also shows that orcas do not seek to be silent, whereas they do when hunting mammals^79–81^. We must acknowledge however that we could not identify callers and more solid conclusions require further investigation.

In conclusion, the fact that visual laterality and exploratory behaviours are sex- and age-dependent has interesting perspectives in both basic (understanding of the underlying, notably psychological, mechanisms and evolutionary processes) and applied research fields. Indeed, human-cetacean encounters are increasing all around the world^82, 83^ and the development of ecotourism over the last 20 years has created a significant pressure on the environment of cetaceans, and this is not limited to cetaceans. Complementary studies are necessary to explore further the different hypotheses raised here, and notably to test to which extent the motivation, the temperament, the degree of attention and the level of experience of animals are determining factors of behavioural variations.

## Materials and Methods

### Study animals, context of the study and video recordings

The presence of orcas in the Kalkfjord (Norway) is directly linked to the massive presence of specific prey, i.e. atlantic herrings (*Clupea harengus*). As a result, the same population of orcas comes back to this location every year^84^. Individuals are identified based on classic criterion for that species, such as the size and the shape of the dorsal fin and the saddle patch as well as bodies scars^85^. Only individuals clearly identified and observed at least twice during the study have been included in the data set, i.e. 60 adult females, 18 adult males, 11 unsexed subadults.

The underwater videos used for the analyses have been made by Pierre Robert de Latour during ecotouristic dives organised in Norway on winters 2014 and 2015 (November to February). The expeditions have been set up from the base M/S SULA and the dives started from a small barge, within a narrow period of daylight (i.e. 10 am–2 pm). Orcas are first observed at a distance, and then the barge is used to approach the animals following the USEA (Undersea Soft Encounter Alliance) method developed by Pierre Robert de Latour. This method is based on the principle of a limited intrusion into the orcas’ space, who can then decide to approach the barge and the divers further or not. The divers (including snorkelers), with a maximum of eight people simultaneously in the water, are gathered just behind the guide, Pierre Robert de Latour. All people are commanded to stay as a group, move slowly and silently.

Video recordings have been made with an underwater camera Sony Ilce A6000 with a Nauticam NA 6000 housing, and equipped with a 24 mm focal equivalent to 24*36 (shutter speed: 1/25 to 1/100, aperture: f2.8, ISO: automatic limited at 3200, White Balance: 4800 k). All in all, 902 video sequences (collected with an *ad libitum* sampling method, i.e. at various times of the diving session and with various durations) have been analysed, that is 540 minutes of film sampled (2014: 323 video sequences lasting 163 minutes in total; 2015: 579 video sequences lasting 377 minutes in total).

Our study is observational and totally non-invasive, based on video scoring. Videos come from the archive of the USEA – Orques Sans Frontieres program (permit #USEA2017-0007).

### Behavioural repertoire and data collection

**Figure 5:**
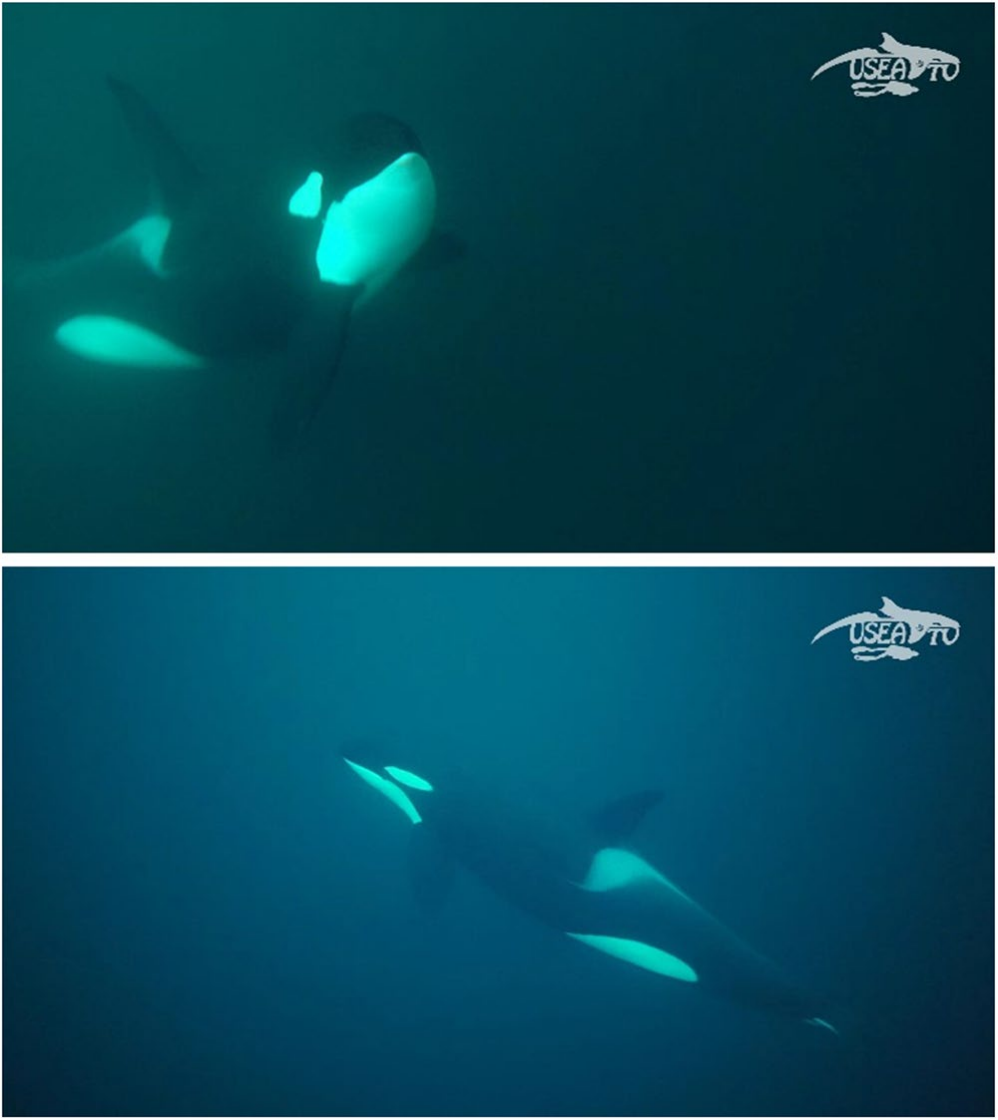
Photos of an adult male (above) and an adult female (below) orcas approaching divers with the respective use of a right and a left eye for visual monitoring (acknowledgments to Pierre Robert de Latour for providing the pictures).

A detailed behavioural repertoire has been established to sample the different movements and other exploratory behaviours of orcas when approaching the divers (see photo examples in Fig. 5). Here, an approach is defined as follows^1^: an approach is systematically an animal movement oriented in the direction of the divers^2^; an approach starts from the moment where an orca enters the camera range and ends as soon as it gets out. The behavioural repertoire, composed of 10 measured variables (most of them being divided into mutually exclusive units), is given in Table 2. For each single approach, we used a 1-0 sampling method to score all measured variables.

**Table 2:** Characteristics of the different types of approach and associated behaviours.

Measured variables	Behavioural unit definitions
**Distance** (minimum distance between the orca and the diving guide reached during a given approach)	The orca approaches the diver from:
	- Very close (less than 5 meters)
	- Close (between 5 and 20 meters)
	- Far (more than 20 meters).
**Angle of approach**	The orca approaches the diver from:
	- The front:
	* from the right side of the diver (angle: 30 to 120°)
	* from the left side of the diver (angle: 200 to 330°)
	* facing the diver (angle: 0°)
	- Below:
	* moving just under the diver
	* arriving vertically from deep water
	- The back.
**Swimming speed**	- Slow: the orca moves but the movements of its caudal fin are hardly detectable
	- Fast: at least one caudal fin movement per second.
**Gaze laterality**	The orca looks at the diver with:
	- its right eye
	- its left eye
	- both eyes (bilateral)
	*For a given approach*, *the eye used may change during the behavioural sequence and each gaze change is counted for the laterality analysis*.
**Gaze duration** (mono- and binocular pooled)	The orca looks at the diver for:
	- less than 6 seconds (Short gaze)
	- more than 6 seconds (Long gaze)
	*For a given approach*, *we measured the total gazing duration*.
**Head movements**	- Horizontal (the orca turns slightly its head towards the diver)
	- Vertical (the orca is facing the diver and moves the head up and down several times in a row).
**Whistle calls**	- Whistling (a high-pitched modulated frequency is heard)
	- Silence (no species-specific sound is heard)
	*It is not possible to confirm whether the caller is the observed orca or a neighbouring one*.
**Belly presentation**	The orca moves the head up presenting its belly to the humans.
**Body rotation**	The orca rotates its body along the horizontal axis.

### Statistical analyses

In a first step, to study the possible existence of a visual laterality bias during approaches at the population level, we compared, using Wilcoxon tests for matched samples, the number of gazes from the left eye with the number of gazes from the right eye directed to the humans based on all individuals sampled in each age-sex class.

In a second step, we tested whether the different individuals differed in the way they approached humans. On the one hand, using Wilcoxon tests for matched samples, we compared, for each measured variable and each age-sex class of individuals, the number of occurrences of the different behavioural units sampled. On the other hand, we calculated for each individual sampled the percentage of expression of a given unit over all the behavioural units of each measured variable. These individual percentages were then used to compare the behavioural profiles of adult males, adult females and unsexed subadults using Kruskal-Wallis and post-hoc Mann - Whitney tests (applying Bonferroni corrections for multiple comparisons). Significant threshold was set at 0.05 (and 0.0167 in case of Bonferroni correction).
